# Psychometric properties of the Jefferson Scale of Empathy: a COSMIN systematic review protocol

**DOI:** 10.1186/s13643-019-1240-0

**Published:** 2019-12-10

**Authors:** Brett Williams, Bronwyn Beovich

**Affiliations:** Department of Community Emergency Health and Paramedic Practice, Monash University Victoria, PO Box 527, McMahons Road, Frankston, Victoria 3199 Australia

**Keywords:** Empathy, Systematic review, Psychometric analysis

## Abstract

**Background:**

Empathy is an important characteristic to possess for healthcare professionals. It has been found to improve communication between professionals and patients and to improve clinical health outcomes. The Jefferson Scale of Empathy (JSE) was developed to measure this quality and has been used extensively, and psychometrically appraised, with a variety of cohorts and in different cultural environments. However, no study has been undertaken to systematically examine the methodological quality of studies which have assessed psychometric factors of the JSE. This systematic review will examine the quality of published papers that have reported on psychometric factors of the JSE.

**Methods:**

A systematic review of studies which report on the psychometric properties of the JSE will be conducted. We will use a predefined search strategy to identify studies meeting the following eligibility criteria: original data is reported on for at least one of the psychometric measurement properties described in the COnsensus-based Standards for the selection of health Measurement INstruments (COSMIN) Risk of Bias checklist, examines the JSE in a healthcare cohort (using the student, physician or health profession versions of the JSE), and is published from January 2001 and in the English language. Conference abstracts, editorials and grey literature will be excluded. Six electronic databases (Medline, EMBASE, PsychInfo, PubMed, Web of Science and CINAHL) will be systematically searched for articles meeting these criteria and studies will be assessed for eligibility by two review authors. The methodological quality of included papers will be examined using the COSMIN Risk of Bias checklist.

**Discussion:**

A narrative description of the findings will be presented along with summary tables. Recommendations for use of the JSE with various cohorts and circumstances will be offered which may inform future research in this field.

**Systematic review registration:**

PROSPERO CRD42018111412

## Background

Empathy is widely considered to be an important attribute for healthcare practitioners and has been empirically shown to improve clinical outcomes [1], and improve communication between caregivers and patients [2]. Evaluating the utility of empathy in healthcare settings is underpinned by the psychometric rigour of the instruments used to measure it. Therefore, it is vital to ensure measurement tools are psychometrically sound.

Empathy is a multidimensional construct and includes both affective and cognitive components. The affective component of empathy involves experiencing the feelings of others [3]. On the other hand, cognitive component is not only the ability to understand the experiences and feelings of others, but also the capacity to communicate this understanding back to them [3]. Although empathy includes these two components, it is viewed predominately as cognitive attribute in healthcare settings [4].

Empathy in healthcare is important because it may enhance positive patient outcomes and satisfaction [1, 5, 6]. It has been reported that empathetic communication also facilitates positive interaction and relationship between patients and the healthcare professionals [6, 7]. Furthermore, this relationship has a positive effect on physicians and can buffer against professional burnout and fatigue [8, 9].

Hojat and colleagues developed the Jefferson Scale of Physician Empathy (JSPE) [3] to measure empathy in healthcare settings. Subsequently, this title was changed to Jefferson Scale of Empathy (JSE) for use in wider cohorts of health profession students and practitioners [10]. For the purposes of this study, “JSE” will encompass all versions of the scale used for various cohorts.

While there are a number of instruments that measure empathy, arguably the JSE is one of most common empathy measurement tools used within the context of healthcare. The instrument has been translated into 55 different languages and used worldwide in countries such as Australia, China, America, Brazil and the Czech Republic, demonstrating its broad use and application [11]. While the JSE has been extensively utilised for the study of empathy in healthcare settings, to the best of our knowledge, no systematic review has been undertaken examining the methodological quality of studies that have assessed psychometric properties of the JSE. The COnsensus-based Standards for the selection of health Measurement INstruments (COSMIN) Risk of Bias checklist [12] is a scale that may be utilised for this purpose. Evaluation of the methodological quality of studies that assess measurement properties of an outcome measurement tool is extremely valuable. It is reasonable that the results and conclusions of studies that demonstrate good methodological quality are able to be used with a higher degree of confidence [13]. The COSMIN Risk of Bias checklist was developed based on consensus of international experts in the fields of psychology, epidemiology, statistics and clinicians [14] and has been widely used in the literature [12]. Using this methodology, the quality of the studies that have evaluated the psychometric elements of the JSE will be assessed, thereby providing a benchmark for the level of confidence with which the different versions of the scale may be used in a variety of settings.

### Development of a scale to measure empathy of healthcare professionals

In 2001, Hojat et al. [3] stressed the need for a psychometrically sound instrument to measure empathy among healthcare professionals and medical students, and developed a scale to measure physician empathy in the USA. Three groups participated in the development study. Group 1 consisted of 55 physicians, group 2 is composed of 41 internal medicine residents and group 3 comprised 193 third year medical students. The first version of the JSE was developed based on an extensive literature review. Subscales from other empathy instruments were then used to test its validity and dimensionality. These included empathetic concern, perspective taking and fantasy scale, adapted from the Interpersonal Reactivity Index (IRI) [15]; warmth and dutifulness, adapted from the NEO Personality Inventory-Revised (NEO-PI-R) [16]; and the Faith in People Scale [17, 18].

The first version of the 90-item JSE was tested using the Delphi method to obtain the content of the instrument and evaluate its face validity and clarity. Group 1 reviewed the first version and deleted items deemed irrelevant to the measurement of empathy. Items were edited to increase clarity and items were added where appropriate. Further validity of the scales was obtained from the 193 third year medical students in group 3. Subsequently, the modified JSE consisted of 45 items, and together with the IRI scale were completed by 41 resident physicians in group 2. Additionally, the modified 45-item JSE together with other instruments (IRI scales, Personality facets of the NEO PI-R, Faith in People Scale, and Personal Attributes) were completed by 193 medical students (group 3).

To investigate the underlying structure of the JSE, data from the medical students underwent factor analysis using the principal components method with orthogonal varimax rotation. Four factors emerged and were retained based on the Kaiser’s criteria, where a component with an eigenvalue above 1 was retained [19]. The first factor that emerged from this study was labelled as ‘Physicians view from the patients perspective’; the second factor was labelled ‘Understanding patients experiences, feelings and clues’; factor three which was reverse scored was labelled ‘Ignoring emotions in patients’; and finally factor four was labelled as ‘Thinking like the patient’. Factors three and four were deemed to be less stable as each factor had less than three items. Among the 20 items that were retained, 17 of these had positive factor structure coefficients with scores ranging from 1 (Strongly Disagree) to 7 (Strongly Agree). The remaining three items with negative factor structure coefficients were reverse scored on a scale ranging from 7 (Strongly Disagree) to 1 (Strongly Agree). Scores range from 20 to 140 with higher score indicating greater empathy.

The criterion-related validity of JSPE has been examined against the IRI. It was found that JSE correlated with the IRI [15, 20]. Moderate correlations were found between perspective taking and empathetic concern (*r* = 0.40). Further, empathetic concern was found with compassionate care (*r* = 0.41) [20]. However, lower correlations were observed between the fantasy and personal distress subscales of the IRI, as these subscales were suggestive of measuring sympathy as opposed to empathy [20].

Subsequent research has proposed various factor structures of the JSE. Many studies have identified a three-factor structure comprising ‘perspective taking’ ‘compassionate care’, and ‘standing in patient’s shoes’ [21–23]. However, other authors have reported other four-factor models. For example, a study on Austrian medical students identified three factors which showed similarities to the aforementioned factors, with an additional factor of ‘negative/no influence of moderating factors and (empathetic) techniques/skills on process/outcome’ [24]. A four-factor model was also proposed in a study of dental students in India, encompassing ‘understanding patient’s feelings’, ‘sense of confusion’ as well as two factors related to ‘ignoring the emotional component’ [25]. A two-factor structure has also been proposed, consisting of ‘perspective-taking’ and ‘compassionate care’ [26]. Given this lack of consistent appraisal of the JSE, a systematic investigation into the quality of assessment of psychometric factors of the JSE is warranted.

### The COnsensus-based Standards for the selection of health Measurement INstruments methodology

The COnsensus-based Standards for the selection of health Measurement INstruments (COSMIN) methodology was developed to guide the performance of systematic reviews of patient-reported outcome measures. A checklist was developed by the COSMIN group to enable the evaluation of the methodological quality of studies on measurement properties with self-reported measurement tools [12], and consists of standards used to assess 10 measurement properties. It should be noted that the COSMIN methodology uses the word ‘patient’ to identify the relevant population; however, this may be modified to suit the population under examination [27]. In the present study, the terms ‘healthcare student’ or ‘healthcare professional’ are the more appropriate terms.

Generally, the COSMIN methodology is employed to make a systematic evaluation of the most suitable tool to measure a certain construct, for example, physical function measures used in intensive care units [28] or health-related quality of life in cervical cancer patients [29]. However, it can also be used to review the measurement properties of a single outcome measurement instrument [27, 30, 31]. In the present review, studies reporting on the measurement properties of the JSE will be examined using the guidelines provided by the COSMIN group and their Risk of Bias checklist [12, 27].

A review of the literature indicated the majority of published papers using the COSMIN methodology utilised a superseded version of the scale [13, 14]. Since this time, the methodology has been updated considerably [12, 27]. For detailed explanation of the changes made to the COSMIN instrument, the reader is encouraged to consult Mokkink et al. [27]. However, the major modifications to the updated version are as follows:

- Inclusion of poorer quality studies in the summary of published studies.
- Removal of criteria regarding a reasonable gold standard for criterion validity and responsiveness. An exception can be made when a shortened instrument is compared with the original long version; the latter being deemed as the gold standard.
- Removal of standards on formulating hypotheses for testing construct validity and responsiveness
- Removal of standards on adequate sample size for single studies from categories where it is possible to pool the results. Sample sizes can then be taken into account when formulating conclusions.
- Indicators of the quality of language translation processes for outcome tools are no longer assessed.
- The ratings for each psychometric characteristic have been changed from ‘excellent’, ‘good’, ‘fair’ and ‘poor’, to the terms ‘very good’, ‘adequate’, ‘doubtful’ and ‘inadequate’, with an additional ‘not applicable’ option.
- Removal of standards on missing data and handling missing data as the lack of reporting on number of missing items and on how missing items are handled does not automatically result in biased results of the study.

The present study utilised the updated version of the COSMIN Risk of Bias checklist [12].

The aim of this study is to undertake a systematic review which will evaluate the methodological quality of reports of psychometric properties of the JSE used to examine empathy in a healthcare cohort, utilising the COnsensus-based Standards for the selection of health Measurement INstruments (COSMIN) Risk of Bias checklist [12]. The measurement properties considered will include the following: outcome measure tool development, content validity, structural validity, internal consistency, cross-cultural validity\measurement invariance, reliability, measurement error, criterion validity, hypotheses testing for construct validity and responsiveness.

## Methods/design

This protocol was developed according to the Preferred Reporting Items for Systematic Reviews and Meta-analyses protocols (PRISMA-P) (see Additional file 1) [32]. The protocol is registered in the PROSPERO database with an identification number CRD42018111412.

The COSMIN Risk of Bias checklist [33] will be utilised to evaluate studies which have assessed the psychometric properties of the JSE. Eligible studies will be identified through a systematic search of the literature. Such an evaluation will demonstrate which aspects of the JSE have been assessed satisfactorily as well as those assessed unsatisfactorily, and enable recommendations to be made regarding the degree of confidence one can bestow on studies which have utilised the JSE.

### Eligibility criteria

Studies will be included if they report original data on at least one of the psychometric measurement properties described in the COSMIN Risk of Bias checklist [12] (see Table 2), examine the JSE in a healthcare cohort (using the student, physician or health profession versions of the JSE), were published from January 1, 2001, and were written in English. Conference abstracts, editorials and grey literature will be excluded.

### Search methods

Six databases (Medline, EMBASE, PsychInfo, PubMed, Web of Science and CINAHL) will be systematically searched for journal articles published from January 2001 to current literature. No benefit will be gained from including an earlier commencement date for the literature search, as information on the JSE has been published from 2001. The search terms will be ‘Jefferson’, ‘Empathy’, ‘Health Personnel’, ‘Student’, ‘Reliability’, ‘Validity’, ‘Factor Analysis’, ‘Classic Test Theory’ and ‘Item Response Theory’. See Table 1 for literature search strategy.

**Table 1:** Search strategy

1.	jefferson AND empathy,
2.	health personnel OR student
3.	reliability OR validity OR factor analysis OR classic test theory OR item response theory
4.	1 AND 2 AND 3

### Screening

Citation details of articles found by the above search strategy will then be exported into an Endnote library and duplicates removed. Two reviewers (BW, BB) will independently assess the titles and abstracts of remaining articles to determine whether they meet the inclusion criteria. Articles remaining after title/abstract screening will then be subject to full-text review by the same two authors using the same eligibility criteria. Any disagreement arising will be resolved through discussion by the two reviewers until a consensus is reached.

### Data extraction

The eligible studies will be examined by one reviewer (BB) and relevant information captured regarding the general characteristics of the studies includes authors, language of use, country in which the study took place, population of study participants, mean age, gender and response rate. Data is to be extracted according to guidelines from the COSMIN Risk of Bias checklist [12]. The other author (BW) will verify data extraction by reviewing approximately 30% of the first author’s assessments.

The term ‘Outcome measure instrument development’ will be used instead of the original ‘Patient reported outcome measure development’ to more accurately reflect that the included studies examined outcomes reported by healthcare professionals or students, as opposed to patients.

### Risk of bias assessment

Assessment of the methodological quality of the selected studies will be carried out using the COSMIN Risk of Bias checklist which contains ten boxes used to assess a study on methodological quality standards (see Table 2).

**Table 2:** COSMIN Risk of Bias checklist summary [12]

Content validity	
Box 1	Outcome measure tool development
Box 2	Content validity
Internal structure	
Box 3	Structural validity
Box 4	Internal consistency
Box 5	Cross-cultural validity\Measurement invariance
Remaining measurement properties	
Box 6	Reliability
Box 7	Measurement error
Box 8	Criterion validity
Box 9	Hypotheses testing for construct validity
Box 10	Responsiveness

Each measurement property is to be scored on a four-point scale using the descriptors ‘very good’, ‘adequate’, ‘doubtful’ and ‘inadequate’. A ‘not applicable’ option is also included for each property, and the measurement properties that are relevant to each study will be assessed. An overall score for the methodological quality of each measurement property will be determined by taking the lowest rating of any of the items in a box, that is ‘the worst score counts’ principle [34]. For example, in the structural validity box, if a confirmatory factor analysis has been undertaken (a ‘very good’ rating), but the sample size was < 5 times the number of items (an ‘inadequate’ rating), the overall quality rating for this box will be judged as ‘inadequate’.

Two reviewers will assess the assessment of the methodological quality of the studies independently. Any disagreement that arises will be resolved by discussion by the reviewers until a consensus is reached.

### Data management and analysis

Endnote (v. 18.2) will be utilised to store citation details of the articles retrieved through the literature search.

A narrative description of the findings will be presented along with summary tables. Recommendations for use of the JSE with various cohorts and circumstances will be offered inform future research in this field.

## Discussion

This systematic review will provide thorough information with regard to the methodological quality of existing studies of psychometric properties of the JSE. Although the JSE has been extensively used in research of empathy in healthcare, to the best of our knowledge, no studies exist which evaluate the methodological quality of the published literature in this area. Thus, the findings of this review will provide important information to researchers on the utility and relevance of this outcome measurement instrument within various cohorts and settings.

## Supplementary information

**Additional file 1.** PRISMA-P 2015 Checklist. Description of data: a checklist to be used with systematic review protocol submissions.

## Data Availability

Not applicable

## Abbreviations

COSMIN: COnsensus-based Standards for the selection of health Measurement Instruments
IRI: Interpersonal Reactivity Index
JSE: Jefferson Scale of Empathy
JSPE: Jefferson Scale of Physician Empathy
NEO-PI-R: NEO Personality Inventory-Revised
PROM: Patient-reported outcome measure
